# Let-7 miRNAs Modulate the Activation of NF-κB by Targeting TNFAIP3 and Are Involved in the Pathogenesis of Lupus Nephritis

**DOI:** 10.1371/journal.pone.0121256

**Published:** 2015-06-25

**Authors:** Jun Liu, Lin Zhu, Guang-liang Xie, Jing-fang Bao, Qing Yu

**Affiliations:** Department of Nephrology, Shanghai General Hospital, Shanghai Jiaotong University, Shanghai, P.R. China; University of Utah School of Medicine, UNITED STATES

## Abstract

TNFAIP3 is a ubiquitin-editing enzyme that negatively regulates multiple NF-κB signaling pathways and dysregulation of TNFAIP3 is related to systemic lupus erythematosus (SLE). Although there exists evidence indicating that microRNAs (miRNAs) modulate the expression of TNFAIP3, whether and how miRNAs regulate TNFAIP3 and contribute to lupus nephritis (LN) is still not well understood. In this study, we screened eleven selected miRNAs that potentially regulated TNFAIP3 expression by dual luciferase assay and found that Let-7 miRNAs repressed TNFAIP3 expression by targeting the 3′UTR of TNFAIP3 mRNA. Overexpression of Let-7 miRNAs led to increased phosphorylation and sustained degradation of IκBα and enhanced phosphorylation of p65 following TNFα stimulation and promoted SeV-induced production of cytokines in HEK293T cells. In addition, the expression of Let-7 miRNAs was significantly up-regulated, and TNFAIP3 level was remarkably down-regulated in samples from LN patients compared control samples. Our findings have uncovered Let-7-TNFAIP3-NF-κB pathway that is involved in LN and thus provided a potential target for therapeutic intervention.

## Introduction

Lupus nephritis (LN) is a kind of kidney disorder caused by systemic lupus erythematosus (SLE), which is a highly complex autoimmune disease. LN contributes to the major cause of morbidity and mortality in patients with SLE, affecting up to 70% of SLE patients [1]. Histological features of LN include increased numbers of mesangial cells, overproduction of extracellular matrix, and infiltration of inflammatory cells, leading to the development of sclerosis and fibrosis [2]. Emerging evidence shows that a large number of cytokines and chemokines were involved in the pathogenesis of LN [3–6].

It has been well recognized that the transcription factor nuclear factor-κB (NF-κB) plays a critical role in regulating the expression of inflammatory cytokines and chemokines. The canonical (p65/p50) and non-canonical (RelB) NF-κB proteins are sequestered in the cytosol by inhibitor of κBα (IκBα) or p100, respectively. Stimulation with inflammatory signals such as TNFα or LPS results in phosphorylation-dependent degradation of IκBα, whereas stimulation with a smaller range of signals such as LTa/b and BAFF leads to processing of p100 to p52, releasing the NF-κB proteins into nucleus. Over activation of NF-κB has been suggested to be involved in human IgA nephropathy, membranous nephropathy, diabetic nephropathy and LN [7–9].

TNFAIP3 (also known as A20) is an ubiquitin-editing enzyme that negatively regulates the activation of NF-κB in various signaling pathways. It has been shown that the expression of TNFAIP3 is reduced in patients with SLE, and nucleotides variants in the enhancer elements of TNFAIP3 have been confirmed to be related to the predisposition of SLE[10]. In addition, there are also evidences indicating that MicroRNAs (miRNAs) modulated the expression of TNFAIP3 [11, 12], while the relation between miRNAs and TNFAIP3 in LN is still not well understood.

miRNAs are short non-coding RNAs which modulate gene expression by binding to the complementary segments present in the 3’UTR of the mRNAs of protein coding genes. Abnormal expression of miRNAs has been found related to many human diseases spanning from psychiatric disorders to malignant cancers[13–15]. Recently, increasing evidence has shown that the expression of a group of miRNAs is disturbed in LN patients and some of them are related to the pathogenesis of LN.

Bidirectional interplays between the NF-κB pathway and miRNAs have recently been illustrated[16, 17]. In this study, we screened 11 selected miRNAs which potentially repressed the expression of TNFAIP3 by dual luciferase assay and found that Let-7 family members specifically targeted the 3’UTR of TNFAIP3 mRNA. In addition, the expression of Let-7 miRNAs was significantly potentiated in sample from LN patients compared to control samples. Conversely, the expression of TNFAIP3 was substantially decreased. Our study hints that Let-7 miRNAs are involved in the pathogenesis of LN by targeting TNFAIP3 and serves as a potential therapeutic target for treatment of LN.

## Results

### The expression of TNFAIP3 was repressed by Let-7 family

**Fig 1:**
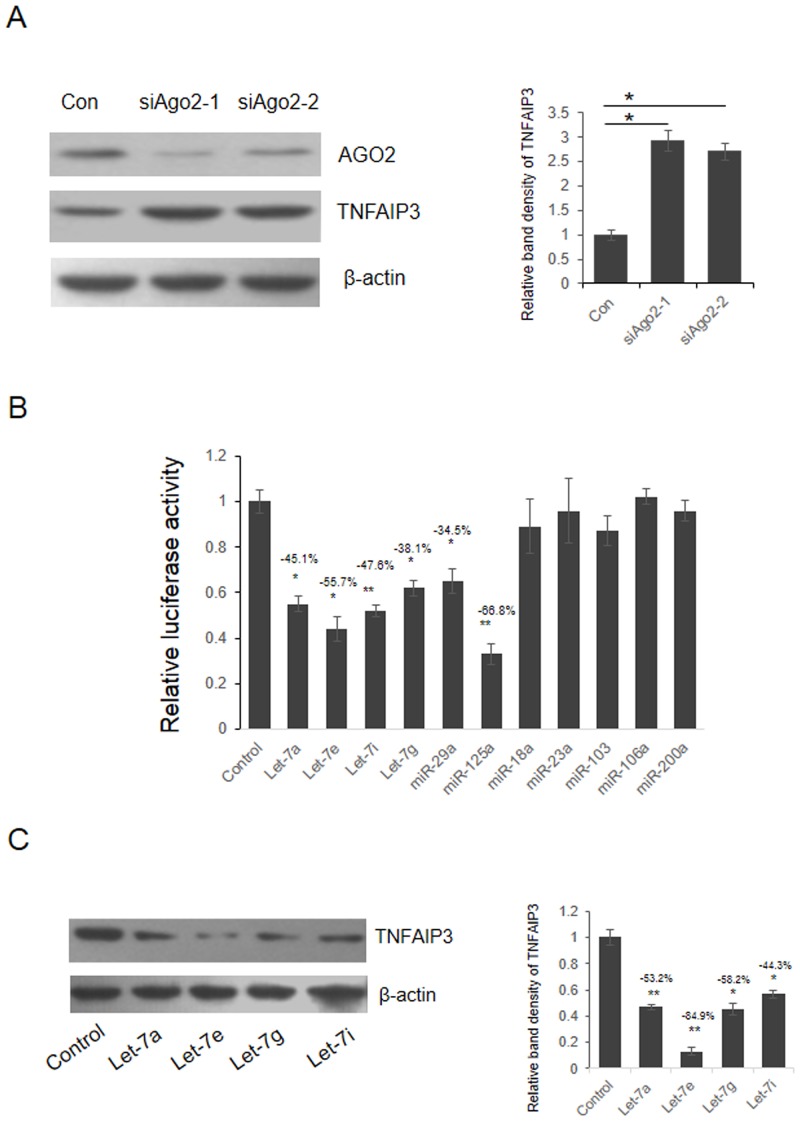
TNFAIP3 expression was repressed by Let-7 miRNAs in HEK293T cells. (A) HEK293T cells were transfected with two AGO2 specific siRNAs, 48 hours after transfection, cells were lysed and the expression of AGO2 and TNFAIP3 was detected by western blot. The band density was quantified by using Quantity One software and the results were analyzed by student’s t-test. *p<0.05, **p<0.01. (B) Luciferase reporter system was employed to screen the miRNAs may repress TNFAIP3 expression. Luciferase vector containing full length TNFAIP3 3’UTR and miRNA mimics were transfected into HEK293T cell. 48 hours after transfection, cells were lysed and luciferase activity was detected. *p<0.05, **p<0.01. (C) HEK293T cells were transfected with Let-7 miRNAs, 48 hours after transfection, cells were lysed and TNFAIP3 was detected by western blot. The band density was quantified by using Quantity One software and the results were analyzed by student’s t-test. *p<0.05, **p<0.01.

To confirm that the expression of TNFAIP3 was repressed by miRNAs, we first suppressed the expression of AGO2, a core component of RNA induced silencing complex (RISC), by AGO2 specific siRNA in HEK293T cells and examined the expression of TNFAIP3. As shown in Fig 1A, along with the significant reduction of AGO2, the expression of TNFAIP3 was up-regulated remarkably, indicating that miRNAs modulate the expression of TNFAIP3. To further identify which miRNA repress TNFAIP3 expression directly, we constructed the TNFAIP3 luciferase reporter vector which containing the full length of 1965bp 3’UTR of TNFAIP3. We screened 11 miRNAs which were predicted to target TNFAIP3 3’UTR directly by TargetScan (http://www.targetscan.org). As shown in Fig 1B, 6 (Let-7a, Let-7e, Let-7i, Let-7g, miR-29c and miR-125a) out of 11 miRNAs repressed the luciferase activity significantly by targeting 3’UTR of TNFAIP3.

To further investigate whether endogenous TNFAIP3 expression was modulated by Let-7 family members, HEK293T cells were transient transfected with Let-7 mimics. As shown in Fig 1C, the expression of TNFAIP3 was remarkably reduced by overexpression of Let-7 miRNAs, especially Let-7e.

**Fig 2:**
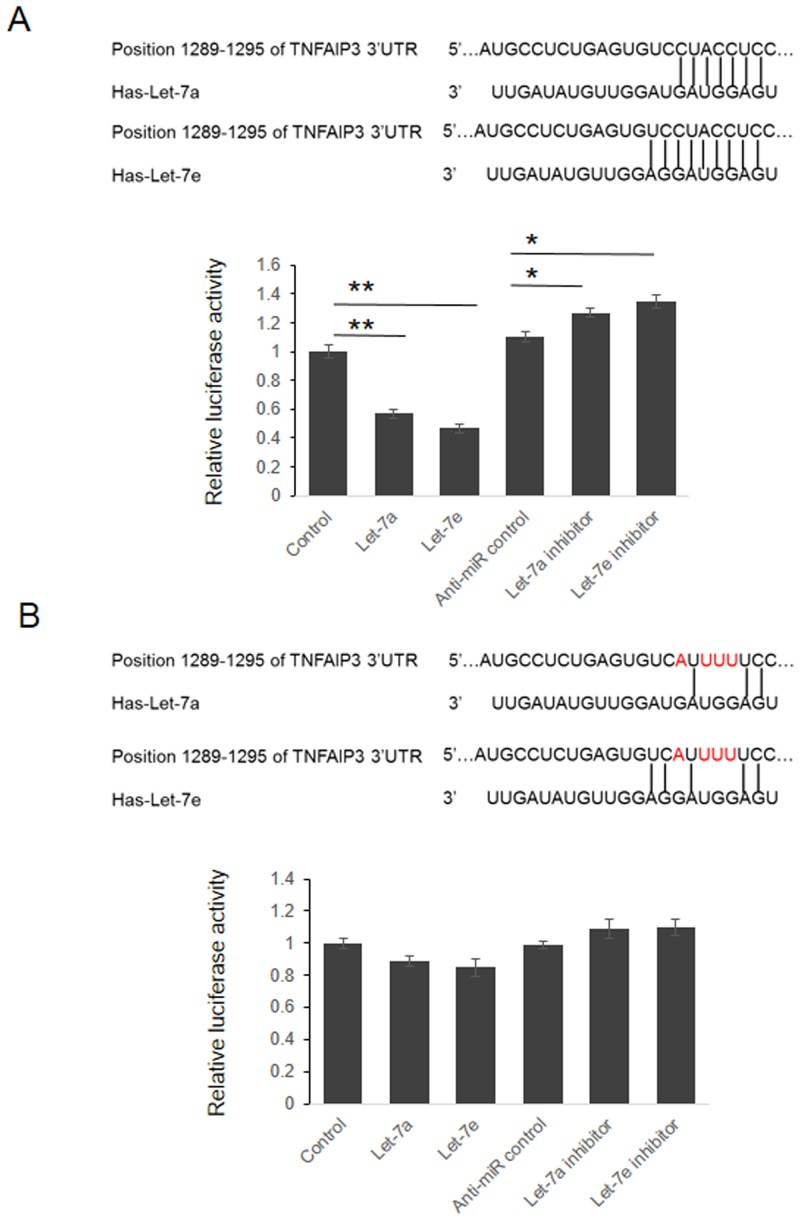
Identify the binding site of Let-7 in TNFAIP3 3’UTR. (A) HEK293T cells were co-transfected with miRNA control, Let-7a/e mimic, anti-miR control or Let-7a/e inhibitor and pGL3-TNFAIP3 for dual-luciferase assay. PRL-TK containing Renilla luciferase was co-transfected for data normalization. (B) The reporter vector containing four nucleotides mutant TNFAIP3 3’UTR was used to confirm the target site of Let-7.*p<0.05, **p<0.01.

We next determined the target sites of Let-7 miRNAs. We made reporter vectors in which the luciferase-coding sequence was followed by mutated 3’UTR of TNFAIP3 mRNA (4 nucleotides mutation in the putative Let-7 binding region) (Fig 2B) and examined the luciferase activity when co-transfection with let-7 miRNAs. The Let-7a and e level in the cells were detected by qRT-PCR (S1 Fig). As shown in Fig 2A, the luciferase activity was reduced about 43.2% or 52.8% by Let-7a or Let-7e (p<0.05), respectively, when luciferase-3’UTR (wild-type) vector was used. In contrast, the luciferase activity of luciferase-3’UTR (mutant) was not significantly reduced by Let-7a or Let-7e (p>0.05) (Fig 2B). These data suggest that Let-7a and Let-7e suppress the expression of TNFAIP3 through binding to seed sequence at the 3’-UTR of TNFAIP3.

### Let-7 overexpression elevates NF-κB activity in HEK293T cells

**Fig 3:**
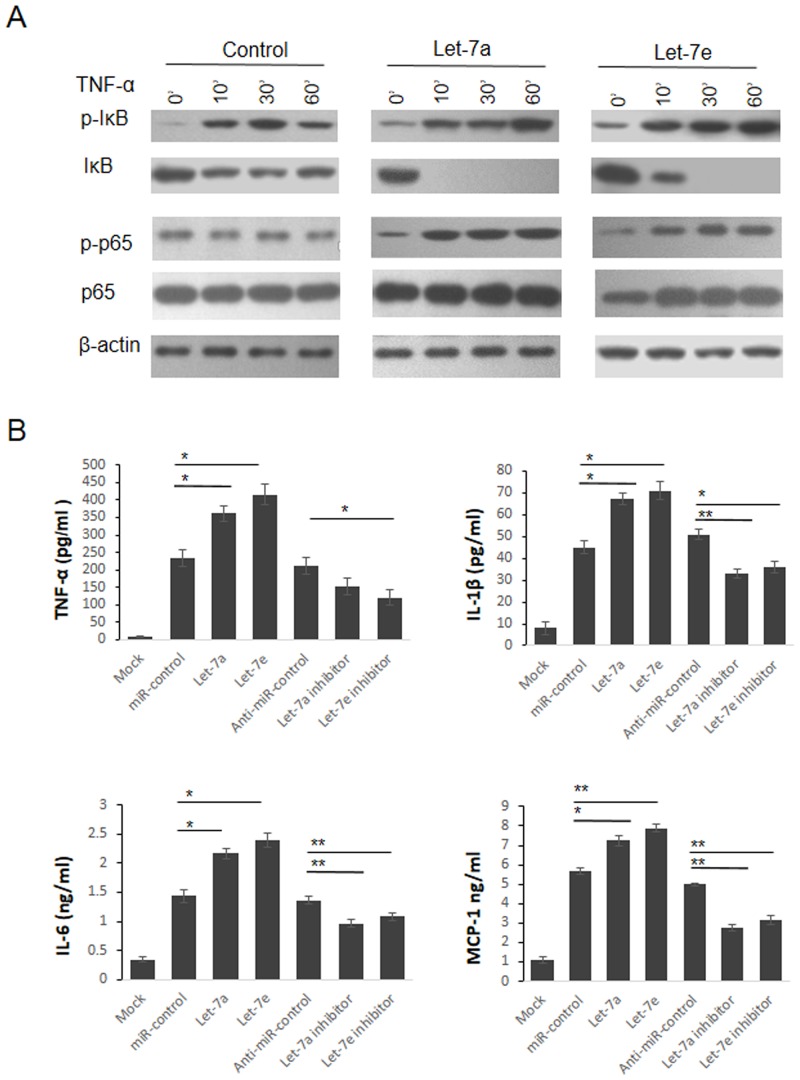
Let-7 activate NF-κB signaling through repressing TNFAIP3 expression. (A)HEK293T cells were transfected with Let-7a or Let-7e, 48 hours later, cells were treated by TNF-α (200ng/ml) for different times. The protein levels of IκB, p-IκB, p65 and p-p65 were detected by western blot with β-actin as loading control. (B) Let-7a or Let-7e was transfected into HEK293T cells. 48 hours after transfection, the cells were washed with PBS and infected with SeV in serum-free medium for 2 hr. 12 hours later, the cell culture supernatants were collected, the levels of TNF-α, IL-6, IL-1β and MCP-1 were detected by ELISA. *p<0.05, **p<0.01.

TNFAIP3 functions as a terminator of the NF-κB activation following cellular exposure to various pathogens or pro-inflammatory cytokines such as TNFα or LPS [18, 19]. Thus, we next examined the effect of Let-7 overexpression on TNFα-induced activation of NF-κB. As shown in Fig 3A, overexpression of Let-7a or Let-7e led to increased phosphorylation and sustained degradation of IκBα, which was accompanied by enhanced phosphorylation of p65 after TNFα stimulation.

Viral infection is another stimulation that activates NF-κB and production of pro-inflammatory cytokines. We next examined the effect of Let-7 on the production of pro-inflammatory cytokines after virus infection. As shown in Fig 3B, SeV infection resulted in robust expression of TNF-α, IL-6, IL-1β and MCP-1, which was significantly up-regulated by Le7-a/e mimics and reduced by Let-7a/e antagonists, respectively. These data suggest that Let-7 miRNAs positively regulate NF-κB activation in response to various stimuli.

### Up-regulated Let-7 miRNAs were accompanied by reduced TNFAIP3 in LN patients

**Fig 4:**
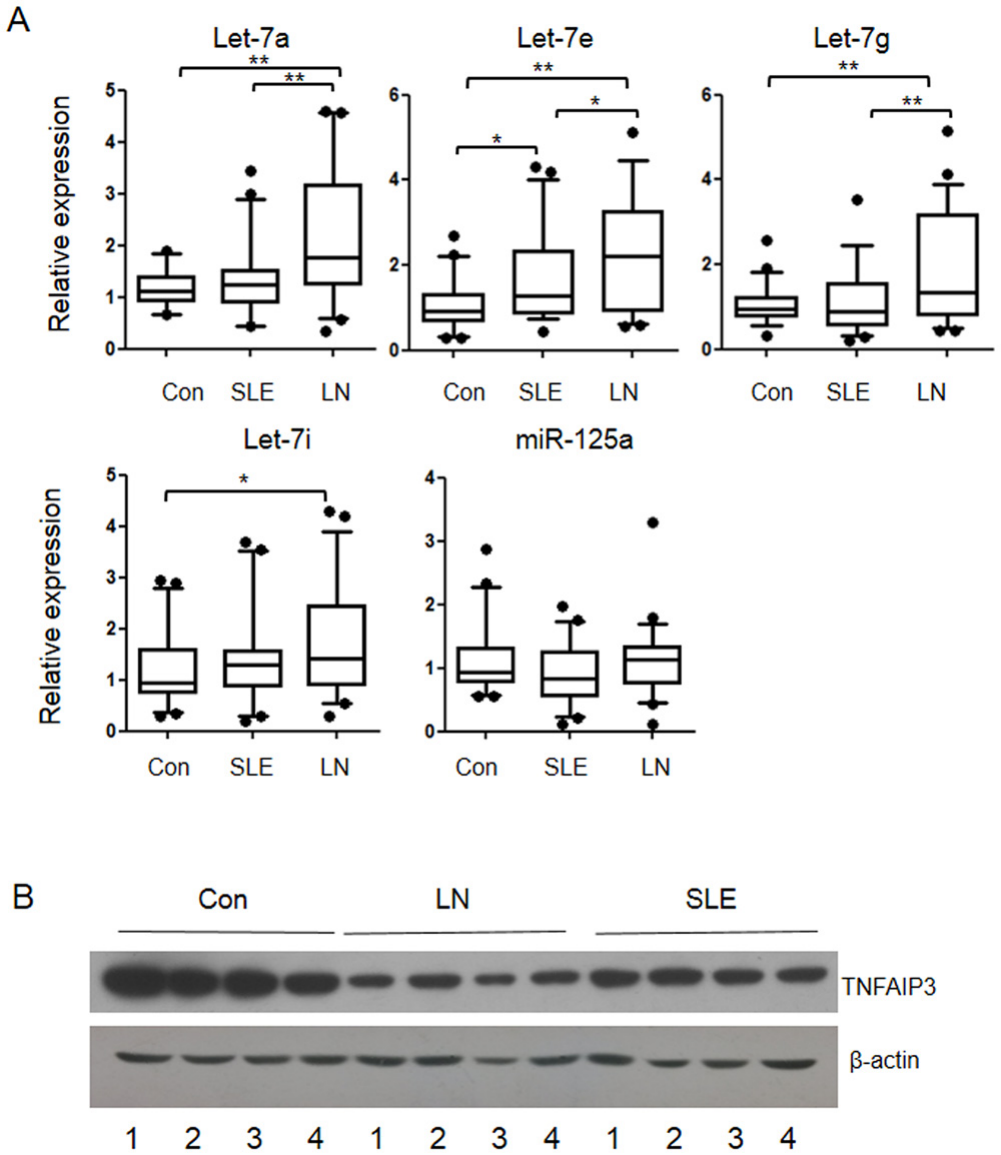
Up-regulated Let-7 miRNAs and down-regulated TNFAIP3 was found in kidney biopsies of LN patients. (A) The expressions of five selected miRNAs in kidney biopsies of LN and SLE patients or normal controls were detected by real time RT-pPCR. Box and whisker plot using Tukey’s method. Lines represent quartiles and individual dots represent outliers. *p<0.05, **p<0.01. (B) The expression of TNFAIP3 in kidney biopsies of LN and SLE patients or normal controls were detected by western blot.

To further explore the expression profiles of Let-7 miRNAs in vivo, we detected their expression in kidney tissue samples of LN patients or normal kidney tissues. The information of the LN patients was exhibited in Table 1. MiR-125a, which has been reported as a suppressor of TNFAIP3, was also detected. As shown in Fig 4A, the expression of Let-7a/e/i/g was significantly up-regulated in the tissue samples of LN patients when compared with normal controls or SLE patients. In contrast, the expression of miR-125a was not significantly changed among these three groups. Conversely, TNFAIP3 protein level was remarkably decreased in the kidney tissues from LN patients compared to samples from normal or SLE patients (Fig 4B). These data together indicate that the up-regulation of Let-7 miRNAs is co-related with down regulation of TNFAIP3, which may contribute to the pathogenesis of LN.

**Table 1:** Clinical and histopathological data of LN patients participating in the study.

Diagnosis and classification	Number	Age (years)	Proteinuria(g/day)	Serum creatinine(μmol/L)
III	5	31.40±5.45	1.57±0.08	93.48±14.23
IV	37	33.84±7.65	3.23±1.06	195.02±102.42
V	9	32.89±9.01	3.71±1.99	176.09±74.43
Total LN	51	33.39±7.58	3.16±1.34	180.64±96.22

Data are expressed as mean±SD.

### Lin28-Let-7 axis modulates the expression of TNFAIP3

**Fig 5:**
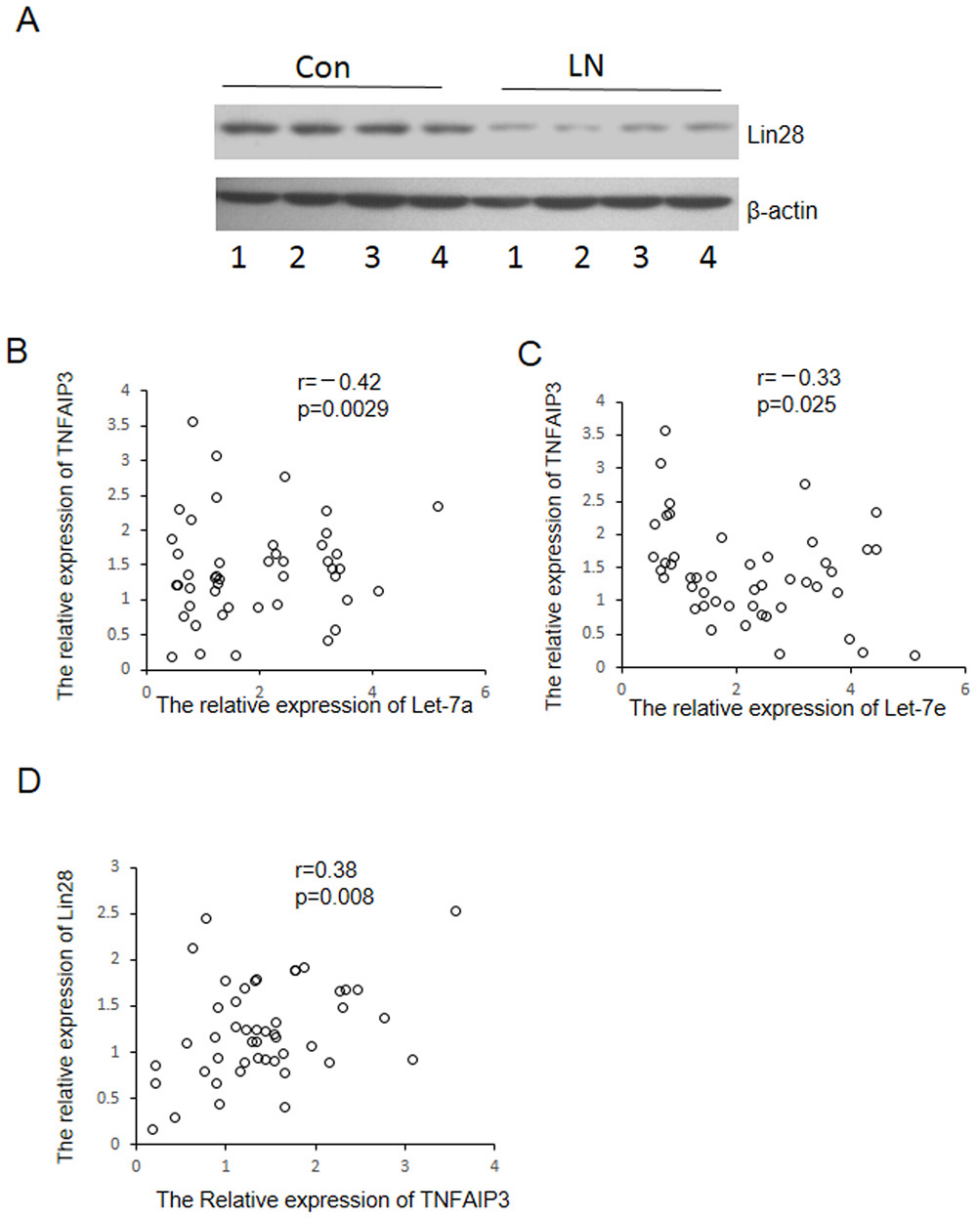
Correlation analysis between Lin28 and TNFAIP3 or Let-7 TNFAIP3. (A) The expression of TNFAIP3 was detected in kidney biopsies of LN patients and normal controls by western blot. The expression of Let-7a (B) and Let-7e (c) in kidney biopsies of LN patients was detected using stem-loop qRT-PCR. The expression of TNFAIP3 and Lin-28 was detected by western blot. To quantify the expression of TNFAIP3 and Lin-28, the band intensity of western blot was semi-quantitatively calculated by using Image J software. And χ2-analysis was used to analyze the correlation among the expression of TNFAIP3, Lin28 and Let-7. *p<0.05, **p<0.01.

Lin28 is an RNA binding-protein which can bind to the Let-7 pre-microRNA and blocks production of the mature Let-7 and plays important roles in development, pubertal maturation and cancer malignancy. Interestingly, the expression of Lin28 was substantially reduced in kidney tissues from LN patients compared to control normal samples (Fig 5A). Furthermore, correlation analysis confirmed the relation between Let-7 miRNAs, TNFAIP3 and Lin28 in LN patients. As shown in Fig 5B and 5C, inverse relationships were existed between the expression of Let-7a and TNFAIP3(r = —0.42, p = 0.0029) or Let-7e and TNFAIP3(r = —0.33, p = 0.025) in LN patients. Meanwhile, Lin28 level was positively correlate with the expression of TNFAIP3(r = 0.38, p = 0.008) (Fig 5D). These results indicate a Lin28-let 7-TNFAIP3 axis whose dysfunction would activate NF-kB and lead to LN.

## Discussion

NF-κB is a transcription factor critically involved in development, immunity, inflammation and tumorigenesis. TNFAIP3 is a ubiquitin-editing enzyme which negatively regulates the activation of NF-κB and its down regulation is related to the pathogenesis of SLE. Although there is evidence indicating that miRNAs modulated the expression of TNFAIP3, the relation between miRNAs and TNFAIP3 in pathogenesis of LN is still not well understood. In this study, we found that miRNAs was essential to control the expression of TNFAIP3, as evidenced by the observations that knockdown of AGO2 up-regulated the expression of TNFAIP3. By dual luciferase assay and point mutation analysis, we confirmed that Let-7 miRNAs repressed TNFAIP3 expression by targeting the 3’UTR of TNFAIP3 mRNA. Consistent with these observations, overexpression of Let-7 potentiated TNFα-induced activation of NF-κB and SeV-induced production of pro-inflammatory cytokines. These results indicate that Let-7 miRNAs promote the activation of NF-κB by repressing the expression of TNFAIP3.

Because down-regulation of TNFAIP3 is related to SLE, we further explored the function of Let-7 miRNAs in LN and found that the expression of Let-7 miRNAs and TNFAIP3 in the kidney samples of LN patients was significantly upregulated or decreased compared with normal controls, respectively. In addition, Lin28, a negative regulator of Let-7 miRNAs, was found to be decreased in kidney samples from LN patients compared to the control samples. These results indicate a Lin28-Let-7-TNFAIP3 axis during the pathogenesis of LN which may serve a potential target for therapeutic intervention.

miRNAs are short non-coding RNAs which modulate gene expression by binding to complementary segments present in the 3’UTR of the mRNAs of protein coding genes. Recent evidences support a role of miRNAs in the pathogenesis of SLE. Chafin CB et al., have reported that Let-7a expression is increased in the mesangial cells of pre-diseased and actively diseased NZB/W mice and increases the production of IL-6 in vitro [20]. The expression of glomerular miR-146a and tubulointerstitial miR-638 is confirmed to correlate with LN clinical severity[21]. In the present study, we found the expression of Let-7 miRNAs were significantly increased in the kidney biopsies of LN patients and provided a direct evidence for Let-7 family members being involved in the pathogenesis of LN.

Several polymorphisms in or near the TNFAIP3 locus were described as being associated with inflammatory autoimmune pathology, including SLE[22, 23], rheumatoid arthritis[24], multiple sclerosis[25] and Type 1 diabetes[26]. TNFAIP3 mRNA was also found to be down-regulated in SLE patients[27]. However, whether there are some relations between disturbed miRNAs and TNFAIP3 reduction in LN patients is still unknown. Herein, our study provided the first line of evidence that overexpressed Let-7 miRNAs played a key role in the autoimmune pathogenesis in LN patients via suppressing TNFAIP3, which shed light on the mechanism of LN pathogenesis.

The correlation between let-7 family members and NF-κB pathway was unveiled by David J. et al.[28]. Their research indicated that ectopic expression of subunits of NF-κB (p50 or p65/RelA) can bind to the let-7a-3 promoter and up-regulated pri-let-7a expression. Meanwhile, NF-κB can up regulate LIN28 expression and then repress the pre-let-7a processing. So LIN28 function as a limitation factor for avoiding NF-κB over activation. In this study, we found that let-7 family members were up-regulated in LN patients and LIN28 level was reduced. Up-regulated let-7 family members repressed the expression of TNFAIP3, which is a crucial suppressor of NF-κB signaling. These results suggest that during the pathogenesis of LN, activated NF-κB signaling up-regulate pri-let-7 family members expression; because LIN28 was reduced, overexpressed pri-let-7 can be processed to become mature let-7 and then promote the NF-κB signaling as a positive feedback.

In conclusion, this study confirmed that Let-7 miRNAs represses TNFAIP3 expression through directly targeting 3’UTR. Lin28 down-regulation is one reason of activated autoimmune response in LN patients via down-regulating TNFAIP3 expression.

## Materials and Methods

### Clinical samples

The Ethics Committee of the Shanghai Jiaotong University Affiliated First People's Hospital approved the use of human renal biopsy tissue for this study. All 98 SLE female patients enrolled in the study. Classify a patient as having SLE if: the patient has biopsy-proven lupus nephritis with ANA or anti-dsDNA or the patient satisfies four of the criteria of the American College of Rheumatology (ACR), including at least one clinical and one immunologic criterion. 51 of these patients met the ACR criteria for LN[29]. All the participants provided written consent to participate in this study and the ethics committees of the Shanghai Jiaotong University, Shanghai General Hospital have approved this consent procedure. Renal biopsies were obtained from patients during the period from 2008–2012, by percutaneous renal biopsy. Strict adherence to the Declaration of Helsinki and the requirements for informed consent was observed in all cases. All patients were not receiving immunosuppressive treatment before diagnostic renalbiopsy.47 samples of normal kidney tissue obtained from nephrectomies performed for treatment of renal cell carcinoma served as normal controls. Biopsies diagnosed as LN were classified according to the criteria defined by the International Society of Nephrology/Renal Pathology Society (ISN/RPS) in 2003[30]. LN disease groups consisted of 5 subjects with focal LN (class III), 37 subjects with diffuse LN (class IV), and 9 subjects with membranous LN (class V). The clinical and histopathological data of LN patients can be found in Table 1.

### RNA extraction and miRNAs detection

Quantitive RT-PCR analysis was used to determine the relative expression level selected miRNAs. Total RNA was extracted from tissues samples by using Trizol Reagent (Invitrogen, Carlsbad, CA, USA) according to the manufacturer’s instructions. The expression level of miRNAs was detected by TaqMan miRNA RT-Real Time PCR. Single-stranded cDNA was synthesized by using TaqMan MicroRNA Reverse Transcription Kit (Applied Biosystems, Foster City, CA, USA) and then amplified by using TaqMan Universal PCR Master Mix (Applied Biosystems, Foster City, CA, USA) together with miRNA-specific TaqMan MGB probes (Applied Biosystems, Foster City, CA, USA). The U6 snRNA was used for normalization. Each sample in each group was measured in triplicate and the experiment was repeated at least three times.

### Cell culture and virus infection

HEK293T cells were cultured in Dulbecco’ s Modified Eagle Medium containing 10% fetal bovine serum (Hyclone, Logan, UT, USA), 100 IU/ml penicillin and 10 mg/mL streptomycin. All cells were maintained at 37°C under an atmosphere of 5% CO_2_.

For Sendai virus (SeV) infection, the cells were washed with PBS and infected with virus in serum-free medium for 2 hr, then washed with PBS and cultured in DMEM supplemented with 10% fetal bovine serum.

### Western blotting

Protein extracts were boiled in SDS/β-mercaptoethanol sample buffer, and 30 μg samples were loaded into each lane of 10% polyacrylamide gels. The proteins were separated by electrophoresis, and the proteins in the gels were blotted onto PVDF membranes (Amersham Pharmacia Biotech, St. Albans, Herts, UK) by electrophoretic transfer. The membrane was incubated with mouse anti-Lin28 monoclonal antibody (Abcam, Cambridge, MA, USA), rabbit anti-TNFAIP3 monoclonal antibody (Abcam, Cambridge, MA, USA), mouse anti-β-actin monoclonal antibody (Santa Cruz Biotechnology Inc., Santa Cruz, CA, USA), mouse anti-IκB monoclonal antibody(Santa Cruz Biotechnology Inc., Santa Cruz, CA, USA), or mouse anti-p65 monoclonal antibody(Santa Cruz Biotechnology Inc., Santa Cruz, CA, USA) for 1 h at 37°C. The specific protein-antibody complex was detected by using horseradish peroxidase conjugated rabbit anti-mouse IgG. Detection by the chemiluminescence reaction was carried using the ECL kit (Pierce, Appleton, WI, USA). The β-actin signal was used as a loading control.

### Dual luciferase assay

To generate 3’-UTR luciferase reporter, the full length of 1993bp ofTNFAIP3 3’-UTR was cloned into the downstream of the firefly luciferase gene in pGL3-Control Vector (Promega, Madison, WI USA). MiRNA mimic and inhibitor were synthesized by GenePharma Co., Ltd (Shanghai, China). pRL-TK containing Renilla luciferase was co-transfected for data normalization. For luciferase reporter assays, HEK293T cells were seeded in 24-well plates. Luciferase reporter vectors were co-transfected with miRNA mimic or inhibitor by using lipofectamine 2000 (Invitrogen, Carlsbad, CA USA). Two days later, cells were harvested and assayed with the Dual-Luciferase Assay (Promega, Madison, WI USA). Each treatment was performed in triplicate in three independent experiments. The results were expressed as relative luciferase activity (Firefly luciferase/Renillaluciferase).

### Statistical analysis

The relationship between the expression of miRNAs and the TNFAIP3 protein was analyzed using χ2-analysis. The differences between independent two groups are analyzed by student’s t-test. The findings were considered to be significant at a P-value <0.05. All statistical analyses were performed using the SPSS v. 16.0 software program (SPSS, Inc., Chicago, IL, USA).

## Supporting Information

S1 Fig Quantification of Let-7a and e level in transfected cells. The Let-7a and Let-7e level in the HEK293T cells for luciferase assay were detected by using qRT-PCR. The results were analyzed by student’s t-test and P<0.05 was considered statistically significant. *p<0.05, **p<0.01.
(TIF)
