# Supplementary figures and images for: Let-7 miRNAs Modulate the Activation of NF-κB by Targeting TNFAIP3 and Are Involved in the Pathogenesis of Lupus Nephritis
Source: PLoS One. 2015 Jun 25;10(6):e0121256. doi: 10.1371/journal.pone.0121256 (PMC4482407; doi:10.1371/journal.pone.0121256)

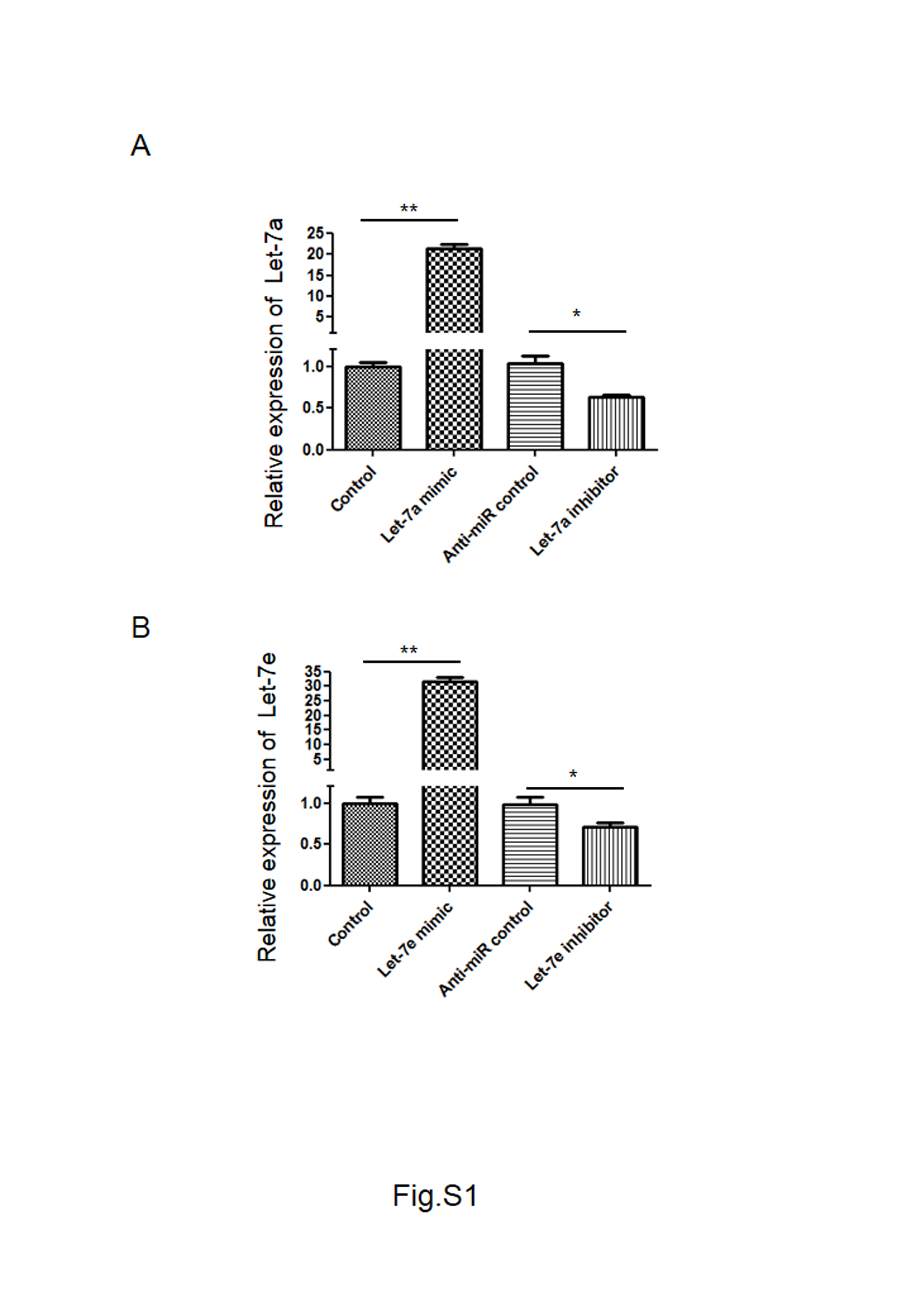

Supplement: S1 Fig — The Let-7a and Let-7e level in the HEK293T cells for luciferase assay were detected by using qRT-PCR. The results were analyzed by student’s t-test and P<0.05 was considered statistically significant. *p<0.05, **p<0.01. (TIF) [file pone.0121256.s001.tif]
